# Large Doses of Ipecacuanha in Acute Dysentery

**Published:** 1871-01

**Authors:** 


Large Doses of Ipecacuanha in Acute Dysentery.—Dr. John Stephens believes in the virtue of the ipecac. in inflammation of the lower bowel, in much larger doses than were formerly given. In an average dose of two scruples, its curative power, he says, is sure and complete; so enthusiastic is he in this that he regards it as much of a specific in acute dysentery as quinine is in intermittent fever. To cases in all degrees of severity, in patients of all ages and conditions, and to any stage of the disease, it is equally applicable. The severe tormina and tenesmus, the frequent bloody discharges often repeated, are symptoms that rapidly abate, and, after two or three doses of the drug, usually are nearly gone.

He gives (as his reported cases indicate) but one dose of the ipecac. each day, preceding it by a dose of opium or laudanum, given a half hour before.

In many cases there occurred no vomiting, and often nothing but nausea. In no case in Dr. S.'s experience has vomiting occurred until the ipecac. had effected its purpose in allaying the disease.

In a part of his cases he painted the abdomen with tincture of iodine or applied sinapisms, but in no case did this lessen the disposition to vomit. He gives the ipecacuanha in a little sweetened water. ---*Medical and Surgical Reporter.*

